# Tuning the magnetic properties of sandwiched hexaferrite/cobalt ferrite exchange-coupled nanocomposites obtained in high-boiling organic solvent

**DOI:** 10.1039/d5ra04855a

**Published:** 2025-09-10

**Authors:** Roy Nygaard, Aleksandr N. Vasiliev, Jianing Chen, Evgeny O. Anokhin, Ekaterina S. Kozlyakova, Maria S. Kondratyeva, Svetlana V. Trusova, Evgeny A. Gorbachev, Lev A. Trusov

**Affiliations:** a Faculty of Materials Science, Shenzhen MSU-BIT University Shenzhen 518172 China ev.a.gorbachev@gmail.com trusov.lev@smbu.edu.cn; b Institute for Nuclear Research of Russian Academy of Sciences Moscow Troitsk 117312 Russia; c Faculty of Chemistry, Lomonosov Moscow State University Moscow 119991 Russia anokhin.evgeny@gmail.com; d Faculty of Physics, Lomonosov Moscow State University Moscow 119991 Russia; e Laboratory of Functional Quantum Materials, National University of Science and Technology “MISIS” Moscow 119049 Russia; f Faculty of Chemistry, Shenzhen MSU-BIT University Shenzhen 518172 China

## Abstract

Exchange-coupled magnetic nanocomposites present significant potential for advanced permanent magnets; however, scalable syntheses that maintain crystallographically coherent interfaces remain challenging. In this study, colloidal Al-substituted strontium hexaferrite nanoplates with average dimensions of 48 nm × 6 nm were covered with epitaxial cobalt ferrite nanolayers *via* the thermolysis of metal acetylacetonates in hexadecane. By simply adjusting the precursor concentration, we create sandwich-like CoFe_2_O_4_/Sr_0.95_Fe_11.5_Al_0.5_O_19_/CoFe_2_O_4_ particles with cobalt ferrite content ranging from 7 wt% to 58 wt%. The results from TEM investigations and theoretical calculations of the energy surface of the interface between CoFe_2_O_4_ and Sr_0.95_Fe_11.5_Al_0.5_O_19_ confirm the existence of a coherent {001} Sr_0.95_Fe_11.5_Al_0.5_O_19_ ‖ {111} CoFe_2_O_4_ interface. Magnetic measurements confirm that the composite particles behave as a single magnetic phase, exhibiting efficient exchange coupling. Magnetic properties reveal a continuous transition from hexaferrite-dominated magnetic behavior to cobalt ferrite-like characteristics as the proportion of the latter increases. This suggests the potential for precise control over the final magnetic properties of the nanocomposite. The proposed synthetic route is gram-scale and yields non-aggregated, uniformly covered nanomagnets with optimal structural and spin coupling between the constituent phases.

## Abstract 2

Thermolysis of metal acetylacetonates in a high-boiling solvent with hexaferrite nanoparticles yields sandwiched hexaferrite/CoFe_2_O_4_ nanocomposites with controlled layer thickness, efficient exchange coupling, and tunable magnetic properties.

## Graphical abstract

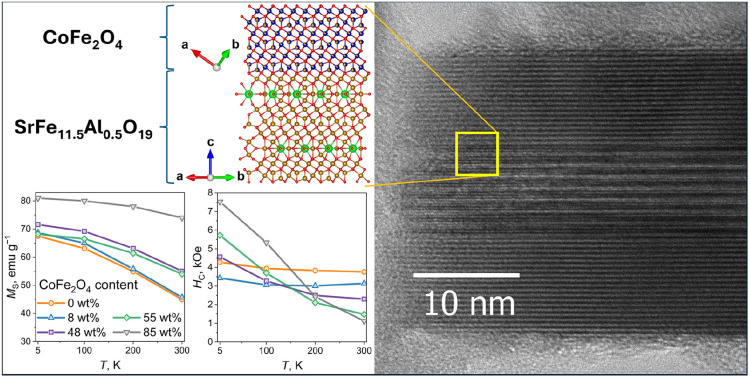

## Introduction

Magnetic nanoparticles play a crucial role across various fields. In medicine, they facilitate targeted drug delivery,^1–3^ diagnostics and therapy,^4^ hyperthermia cancer treatment,^5,6^ and advanced imaging techniques.^2^ In technology, nanoparticles are utilized in the production of data storage devices,^3^ electromagnetic shielding covers,^7^ catalysts,^8^ and sensors.^9^ Their unique ability to respond to external magnetic fields renders them valuable for a wide range of applications, from environmental uses such as water purification and pollutant removal^3^ to magneto-optical applications such as the production of magnetoactive ferrofluids.^10–13^

To meet the demands of most modern advanced applications and tackle future challenges, magnetic nanoparticles must satisfy several essential criteria. Firstly, they should maintain non-zero coercivity and remanence down to nanoscale sizes without transitioning into a superparamagnetic state. Secondly, these nanoparticles must exhibit chemical, mechanical, and thermal stability to ensure their prolonged effectiveness in various conditions or compatibility with sensitive biological environments. These requirements significantly narrow the pool of potential candidate materials for producing magnetic nanoparticles suitable for practical applications. Metallic alloy nanoparticles, such as FePt, FeCo, and Nd–Fe–B, demonstrate exceptional magnetic properties across a broad range of temperatures and particle sizes;^14^ however, they are prone to oxidation and other forms of chemical degradation. In contrast, magnetic oxides with a spinel structure, including γ-Fe_2_O_3_, Fe_3_O_4_, and CoFe_2_O_4_, offer satisfactory magnetic properties along with superior chemical stability compared to metallic nanomagnets. Nevertheless, they experience a transition to superparamagnetic state when their particle size is reduced to several tens of nanometers.^15^ Ultimately, only two types of compounds show promise for full-scale implementation in industry and medicine as permanent nanomagnets: ε-Fe_2_O_3_ and M-type hexaferrites (MFe_12_O_19_; where M = Sr, Ba). While ε-Fe_2_O_3_ nanoparticles exhibit some of the highest coercivity values at room temperature (>20 kOe),^16^ reliable mass-production synthesis methods are still under development. Current synthesis techniques yield limited quantities and offer poor control over the phase composition of the final product.^17,18^ On the other hand, M-type hexaferrites have been extensively studied over the decades, revealing that their nanoparticles possess high coercivity, chemical stability, and a highly anisotropic platelike shape, which leads to additional effects that enhance their utility in magnetic recording, magneto-optics, and magneto-mechanics.^19^

Numerous synthesis routes have been developed to produce single-phase, highly crystalline hexaferrite nanoparticles with excellent magnetic properties, and the glass-ceramic technique is among them. Compared to other synthesis routes, this method has a distinct advantage, allowing the synthesis of highly crystalline, non-aggregated nanoparticles ready for use as nanomagnets or for the manufacture of various nanostructures and nanocomposites. Furthermore, it is scalable, allowing for mass production, and provides opportunities to modify particle shape and size,^20^ as well as to perform various chemical substitutions that further enhance the magnetic properties of the nanoparticles.^21^ Thus, the M-type hexaferrite nanoparticles produced *via* the glass-ceramic technique hold significant practical importance, underscoring the value of research dedicated to the design of composite nanomagnets based on these materials. A promising strategy to further modify and tune their magnetic properties is exchange coupling with other magnetic compounds, creating a nanocomposite based on a hexaferrite cores.^14,22^ Such coupling can result, for example, in an increase in maximum energy product (BH)_max_ or an improvement in the temperature dependence of the magnetic properties. Crucially, the interface must be epitaxial and coherent to ensure strong exchange across only a few nanometers.

Recently, we employed thermolysis of metal–organic salts in a high-boiling solvent to modify the surface of hexaferrite nanoplates by magnetic spinel phases CoFe_2_O_4_ and Fe_3_O_4_,^23,24^ presenting an alternative to co-precipitation from an aqueous solution.^25,26^ The described method allows each hexaferrite nanoparticle to be coated individually with epitaxial layers of spinel phases, resulting in colloids of composite nanomagnets. The combination with cobalt ferrite has resulted in a significant increase in (BH)_max_, especially at low temperatures; however, only composites with a high content (about 85 wt%) of CoFe_2_O_4_ have been described. Herein, we demonstrate that by systematically varying precursor concentration during the thermal decomposition in hexadecane, we can tune the spinel phase content and thereby change the thickness of the spinel layers in the sandwich composite. We provide a detailed study of the magnetic properties of the composites depending on the phase ratio and temperature. Therefore, the specific aim of this study is to establish a scalable, single-step route for producing epitaxial hexaferrite/cobalt ferrite sandwiched nanocomposites and to determine how nanometer-scale variations in the CoFe_2_O_4_ shell thickness influence (i) lattice strain at the hard/soft interface and (ii) the resulting coercivity, saturation magnetization and maximum energy product.

## Experimental

### Materials

The following reagents were used for samples preparation: strontium carbonate SrCO_3_ (≥99.9%, Aldrich), sodium bicarbonate NaHCO_3_ (≥99%, Sigma-Aldrich), iron(iii) oxide Fe_2_O_3_ (<5 μm, ≥99.9%, Sigma-Aldrich), aluminum(iii) oxide Al_2_O_3_ (≥99.9%, Sigma-Aldrich), boric acid H_3_BO_3_ (≥99.8%, Sigma-Aldrich), oleic acid C_17_H_33_COOH (90%, Sigma-Aldrich), iron(iii) acetylacetonate Fe(C_5_H_7_O_2_)_3_ (≥97%, Sigma-Aldrich), cobalt(ii) acetylacetonate Co(C_5_H_7_O_2_)_2_ (≥97%, Sigma-Aldrich), hexadecane C_16_H_34_ (≥98,0%).

### Hexaferrite nanoparticles synthesis

Non-sintered strontium hexaferrite nanoparticles were obtained by the borate glass crystallization method described in detail in our previous work.^27^ Briefly, a glass with a nominal composition of 4Na_2_O–9SrO–5.5Fe_2_O_3_–4.5Al_2_O_3_–4B_2_O_3_ was prepared by melting a 5 g batch of starting materials (SrCO_3_, NaHCO_3_, Fe_2_O_3_, Al_2_O_3_, and H_3_BO_3_) in a platinum crucible in a high-temperature furnace. The mixture was heated up to 1250 °C with a rate of about 10 °C min^−1^ and then exposed to that temperature for 1 hour. The resulting melt was quenched between two rotating steel rollers into the water bath to form glassy flakes.

The obtained glass was isothermally annealed at 700 °C for 2 h to crystallize hexaferrite nanoparticles. During crystallization of the glass, various borate phases (Sr_3_B_2_O_6_, NaSr_4_(BO_3_)_3_, Al_4_B_2_O_9_) are formed, as well as Al-substituted strontium hexaferrite (SrFe_12−*x*_Al_*x*_O_19_) as the only iron-containing crystalline phase. The resulting glass-ceramic material was ground in an agate mortar. The obtained powder was treated with 3% hydrochloric acid to dissolve the borate matrix and form a colloidal solution. After the hydrochloric acid was added to the powder, the mixture was sonicated for 10 minutes with simultaneous heating to 50 °C. Then the magnetic particles were separated by centrifugation (12 k rpm, 20 min), and the remaining powder was washed two times in the acid solution until the non-magnetic matrix was completely removed. The precipitate obtained after that was washed with distilled water. Then the particles were separated by centrifugation, and the powder was dried in the drying box for 30 minutes at 120 °C. The obtained raw hexaferrite particles are labelled as SF (strontium hexaferrite) in this manuscript.

### Hexaferrite/cobalt ferrite composites synthesis

Three composite samples with different hexaferrite to cobalt ferrite ratios were prepared. The samples were labelled CF41, CF21, and CF11 corresponding to the theoretical layer thickness ratios *h*(SrFe_12−*x*_Al_*x*_O_19_) : *h*(CoFe_2_O_4_) = 4 : 1, 2 : 1, and 1 : 1 estimated from the nominal content of cobalt ferrite. Since each composite particle is expected to have two layers of cobalt ferrite formed on both sides of the hexaferrite plate-like nanoparticles, the volume fraction of cobalt ferrite is twice as high as the ratio of the thicknesses of the layers (*i.e.*, 2 : 1, 1 : 1, and 1 : 2, respectively).

The synthesis of the cobalt ferrite layers on the surface of the hexaferrite nanoparticles was carried out by simultaneous thermal decomposition of iron(iii) acetylacetonate Fe(C_5_H_7_O_2_)_3_ and cobalt(ii) acetylacetonate Co(C_5_H_7_O_2_)_2_ under an inert atmosphere in hexadecane acting as a high-boiling solvent (boiling point at 287 °C).^24,28^ The obtained strontium hexaferrite powder SF (100 mg for each sample) and a mixture of iron and cobalt acetylacetonates (151 and 55 mg, 301 and 110 mg, 602 and 219 mg for samples CF41, CF21, and CF11, respectively) were added to 38 ml of hexadecane and 2 ml of oleic acid. The mixture of these components was sonicated for 30 min at room temperature to disperse the particles, then was placed to the three-neck flask (the necks for thermocouple, Ar inlet, and mechanical stirrer). Argon flow of 150 ml min^−1^ for 30 min was used to remove air from the flask, and then it was kept during the synthesis. After that, the solution was heated to 270 °C and was exposed at this temperature for 30 min with continuous stirring. Then the flask with the reaction mixture was quickly cooled to room temperature, after which the Ar flow was stopped.

The composite particles were magnetically separated from the reaction mixture, and then the powder was washed several times alternately with increasing polarity: hexane, acetone, ethanol, 1 M sodium hydroxide solution, and distilled water until the behavior of the powder changed to hydrophilic to remove surfactant residues. After the final wash, the particles were dried in a drying oven at 120 °C.

### Characterization methods

Powder X-ray diffraction (XRD) analyses were conducted using a Rigaku D/MAX 2500 diffractometer (Tokyo, Japan) with Cu Kα radiation. The full-profile analysis of the patterns was carried out by the Rietveld method using MAUD software (ver. 2.9999).^29^ The instrumental line broadening parameters were obtained using the LaB_6_ powder standard. The line broadening study was performed using the “anisotropic no rules” model. In the analysis of peak broadening for nanocomposite samples, the hexaferrite particle size and microstrain were set to the values determined for the initial hexaferrite powder.

Inductively coupled plasma mass spectrometry method (ICP-MS) was used to determine the chemical composition of the raw hexaferrite powder. The analysis was carried out using a PerkinElmer (Waltham, MA, USA) Avio 200 instrument. The sample was dissolved in aqua regia for the analysis.

For transmission electron microscopy (TEM) investigation, a tiny amount of the powder sample was dispersed in ethyl alcohol, and then one drop of suspension was deposited onto a carbon film supported by a copper grid. Transmission electron microscopy was performed using 200 kV field emission microscope JEOL (Tokyo, Japan) 2100 F in a bright-field mode. To determine the average hexaferrite particle diameter, more than 1200 particles were counted, and for the particle thickness, more than 300 particles. Mean particle dimensions and standard deviations were obtained by approximating TEM histograms with a lognormal distribution function.

Magnetic measurements in the maximum field strength of 30 kOe and at temperatures of 5, 100, 200, and 300 K were carried out using a vibrating sample magnetometer (VSM) Cryogenic CFMS-9T (London, United Kingdom). Powder samples were fixed with polymer varnish to avoid their movement in the magnetic field. The values of mass magnetization and coercivity were determined with estimated errors of 0.1 emu g^−1^ and 50 Oe, respectively.

## Results and discussion

**Fig. 1:**
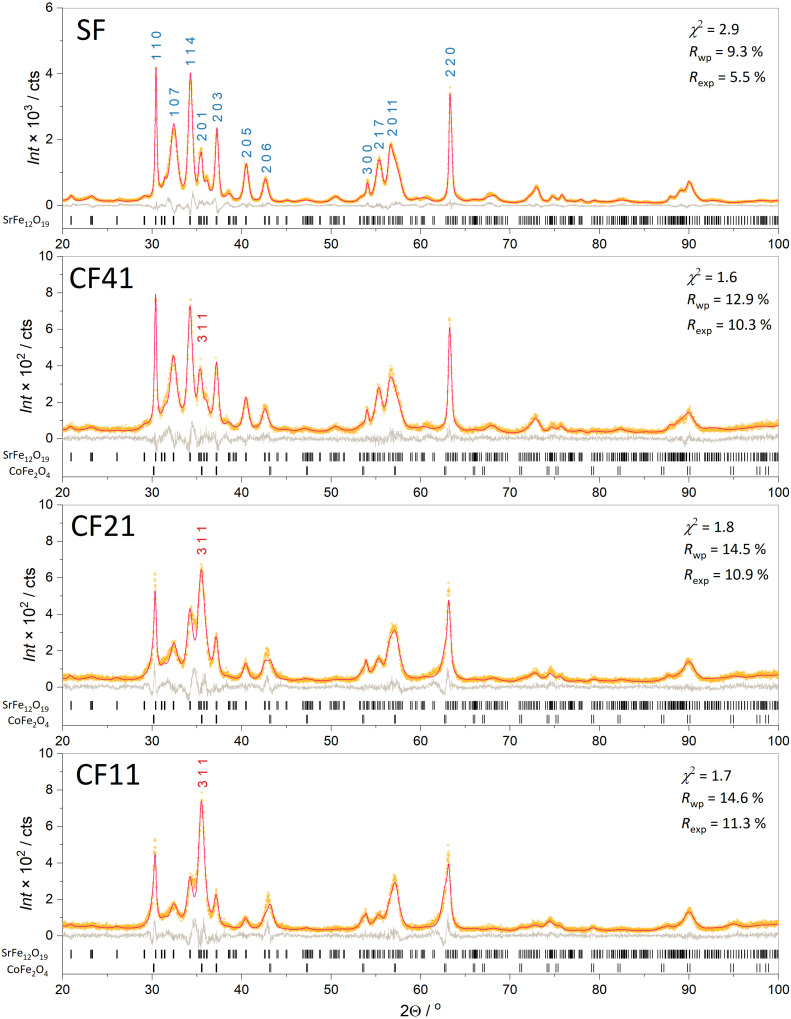
XRD patterns of initial hexaferrite nanoplates and hexaferrite/cobalt ferrite nanocomposites with different compositions: experimental patterns (yellow), theoretical patterns (red), and difference (grey). The discrepancy parameters are also presented. Miller indices for the most intense peaks of hexaferrite are marked with blue numbers, and the strongest peak of cobalt ferrite is marked in red.

According to XRD (Fig. 1), the bare hexaferrite powder (sample SF) represents a single-phase M-type hexaferrite (space group *P*6_3_/*mmc*) with the unit cell parameters *a* = 5.8779 (5) Å and *c* = 23.013 (4) Å. The parameters are slightly reduced in comparison with undoped SrFe_12_O_19_ (*a* = 5.885 and *c* = 23.05 (3) Å),^30^ due to a substitution of some iron ions by aluminum,^31^ which has a smaller ionic radius (*r*^VI^(Fe^3+^) = 0.645 Å and *r*^VI^(Al^3+^) = 0.535 Å).^32^ Since it is known that the cell parameters for the SrFe_12−*x*_Al_*x*_O_19_ solid solution closely follow Vegard's rule and vary linearly with *x*,^33^ the degree of substitution *x* can be estimated as 0.4. The particle composition, also determined by ICP-MS, corresponds to Sr_0.95_Fe_11.5_Al_0.5_O_19_, which is in good agreement with the estimation. It is worth noting that a reduced strontium content in nanoparticles, compared with the stoichiometric value of Sr : Fe = 1 : 12, was also reported earlier for nanoparticles obtained under similar conditions;^27,31,34^ this may be due to the leaching of strontium ions from the surface of the nanoparticles during their extraction from glass ceramics with hydrochloric acid. It is also known that for hexaferrite nanoparticles, the structure with an external spinel structural block is more stable, so the lack of strontium may be associated with a smaller number of strontium-containing layers.^35,36^

A noticeable broadening of the diffraction lines corresponds to the small size of the particles; a much stronger broadening is observed for the hk0 lines (Fig. 1) which indicates the plate-like shape of the particles with a smaller dimension along the crystallographic direction *c* (the thickness *h*) and a larger dimension within the *ab* plane (the diameter *d*). This shape is typical for hexaferrite nanoparticles and follows the symmetry of the crystal structure.^27,37,38^ The analysis of the line broadening using the Rietveld method gave the mean particle diameter *d* = 50.0 nm and thickness *h* = 5.4 nm. In addition, a noticeable anisotropy of microstrain was detected. The RMS microstrain in the lateral directions of the hexaferrite particles (*e.g.*, [100] and [110]) is 0.0005, significantly lower than 0.003 along the [001] direction, which also reflects their plate-like shape.

**Fig. 2:**
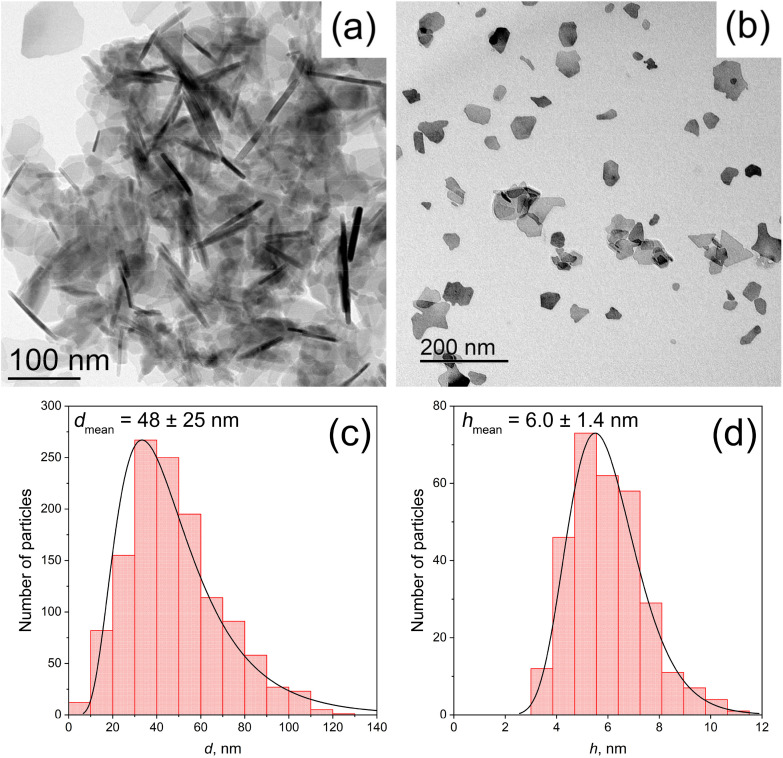
(a) and (b): TEM microphotographs of bare Sr_0.95_Fe_11.5_Al_0.5_O_19_ nanoplates; (c) and (d): corresponding distributions of particle diameters *d* and thickness *h* fitted by log-normal distribution function.

The morphology of the raw hexaferrite particles was also determined using transmission electron microscopy (TEM). Fig. 2a and b depict the same specimen (SF) imaged at two prevailing orientations. In Fig. 2a, many nanoplates are oriented edge-on, providing a side view, whereas in Fig. 2b the plates lie flat on the carbon grid, revealing their lateral morphology. According to TEM, SF particles are thin anisotropic plates with an average diameter of 48 nm and an average thickness of 6 nm (Fig. 2c and d), which is consistent with the estimation given by the Rietveld analysis.

The X-ray diffraction patterns of the hexaferrite/cobalt ferrite composites are also shown in Fig. 1. With an increase in the nominal content of cobalt ferrite phase, its peak 311 (the strongest line of CoFe_2_O_4_) becomes more pronounced, indicating an increase in the proportion of cobalt ferrite in the samples. The Rietveld refinement enabled us to estimate the weight fractions of hexaferrite and cobalt ferrite in the prepared samples and refine the lattice parameters for both phases (Table 1). For samples CF41, CF21, and CF11, the content of the spinel phase was 8, 48, and 55 wt%, correspondingly. The mass fractions obtained by the quantitative X-ray phase analysis are in good agreement with the chemical analysis (EDX, Table 1), which resulted in 7, 50, and 58 wt% of cobalt ferrite for CF41, CF21, and CF11 samples. As can be seen, the proportion of cobalt ferrite in the CF41 sample is noticeably less than the nominal one, which indicates difficult crystallization at a low concentration of the initial solution. Below, for definiteness, we will refer to mass fractions obtained by the EXD method. Chemical analysis also allows us to evaluate the composition of the spinel phase by comparison with the original hexaferrite sample. In sample CF41, the spinel phase is close to CoFe_2_O_4_, and in samples CF21 and CF11, there is an excess of iron, *i.e.*, the composition can be represented as Co_1−*y*_Fe_2+*y*_O_4_ (*y* = 0.4 and 0.3). This suggests a partial reduction of Fe^3+^ to Fe^2+^, which is possible due to the reducing conditions during decomposition of the precursors.

**Table 1:** XRD and EDX results for the initial hexaferrite powder and the composite samples. Chemical compositions are normalized to Sr content. The composition of the cobalt ferrite (spinel) phase is estimated from the chemical analysis (EDX). Cobalt ferrite (spinel) weight fraction *ω* is estimated from the chemical analysis (EDX) and by the Rietveld refinement of the diffraction patterns (XRD). The nominal phase ratio corresponds to the initial solution during the synthesis and represents the maximum theoretical content of the cobalt ferrite (assuming the densities of both phases to be approximately 5 g cm^−3^)

Sample	Sr_0.95_Fe_11.5_Al_0.5_O_19_		CoFe_2_O_4_	Sr : Fe : Al : Co atomic ratio (EDX)	Estimated composition of the spinel phase	*ω* (spinel), wt% (EDX)	*ω* (spinel), wt% (XRD)	*ω* (spinel), wt% (nominal)
	*a*, Å	*c*, Å	*a*, Å					
SF	5.8779 (5)	23.013 (4)	—	1 : 12.1 : 0.7 : −	—	—	—	—
CF41	5.8780 (4)	23.012 (4)	8.380 (9)	1 : 12.8 : 0.8 : 0.3	CoFe_2_O_4_	7 (2)	8 (5)	33
CF21	5.8857 (7)	22.964 (8)	8.369 (5)	1 : 24.1 : 0.9 : 2.7	Co_0.6_Fe_2.4_O_4_	50 (2)	48 (5)	50
CF11	5.8861 (8)	22.949 (9)	8.371 (3)	1 : 26.7 : 0.8 : 4.8	Co_0.7_Fe_2.3_O_4_	58 (2)	55 (5)	66

**Fig. 3:**
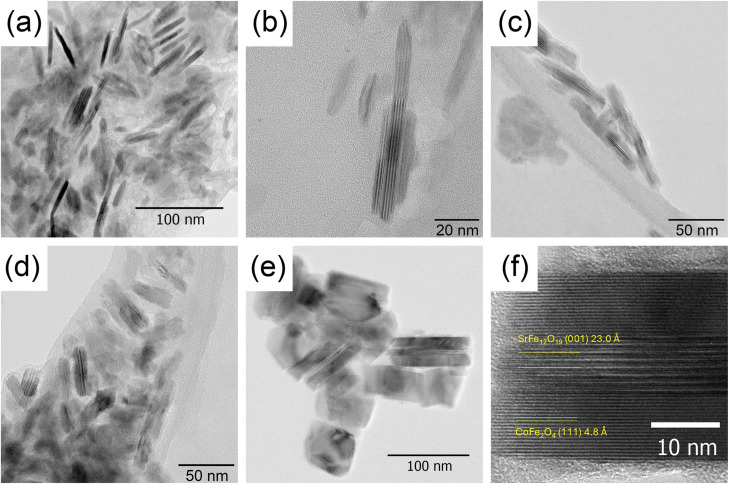
TEM microphotographs of composite Sr_0.95_Fe_11.5_Al_0.5_O_19_/Co_1−*y*_Fe_2+*y*_O_4_ nanoparticles with different weight fractions of Co_1−*y*_Fe_2+*y*_O_4_: CF41 – (a) and (b); CF21 – (c); CF11 – (d and e); side view of the sandwich-like particle of sample CF11 – (f).

Transmission electron microscopy (TEM) showed that the particles in all composite samples retained a lamellar morphology (Fig. 3). Also, no free spherical nanoparticles were detected, which should be formed by cobalt ferrite under the synthesis conditions used.^23^ Thus, it can be assumed that cobalt ferrite was formed mainly on the surface of the original hexaferrite particles. Assuming that cobalt ferrite is distributed uniformly over the both sides of hexaferrite platelets with mean thickness of 6 nm and considering the mass fractions and crystallographic densities (5.0 g cm^−1^ for hexaferrite and 5.3 g cm^−1^ for cobalt ferrite) of the phases, the mean thicknesses of the spinel outer layers can be estimated as 0.2 nm, 2.8 nm, and 3.9 nm for CF41, CF21 and CF11 samples, correspondingly. Indeed, the samples CF21 and CF11 exhibit a pronounced sandwich-like microstructure, while very thin layers in the sample CF41 are very difficult to detect. Cobalt ferrite grows symmetrically on both basal sides (*i.e.*, 001 facets) of the hexaferrite plates and is not found on the lateral surfaces. This indicates that structural conformity is a key factor for hexaferrite particles to act as seeds for cobalt ferrite growth. As shown earlier, the spinel layers are continued with the hexaferrite structure in such a way that the 〈111〉 axis of the spinel phase coincides with the 〈001〉 axis of the hexaferrite,^23,24^ and this is also confirmed by the side view of the particles (Fig. 3e).

A crucial point for our further discussion is the evolution of the lattice parameters of core Sr_0.95_Fe_11.5_Al_0.5_O_19_ as the weight fraction of the spinel phase increases. It is evident that with the rising amount of cobalt ferrite, the parameter *a* of the hexaferrite phase steadily increases. Moreover, the parameter *c* decreases simultaneously, indicating that the hexaferrite lattice is being stretched in the *ab*-plane. This behavior provides a fingerprint of the coherent structural coupling at the interface between these two phases. Conversely, the lattice parameter of Co_1−*y*_Fe_2+*y*_O_4_ remains relatively constant (within the error margin) and fluctuates around its bulk value of 8.373 Å.^40^ Since the EDX results show that the composition of the spinel phase at high concentrations deviates from the ideal stoichiometry Co : Fe = 1 : 2 and shows an increased iron content (*y* > 0), this should lead to an increase in the bulk lattice parameter. This means that the cobalt ferrite is actually compressed by the force exerted by the hexaferrite substrate. These findings are fully consistent with the theoretical calculations of the {001}(Sr_0.95_Fe_11.5_Al_0.5_O_19_)‖{111}(Co_1−*y*_Fe_2+*y*_O_4_) interface that will be discussed below.

**Fig. 4:**
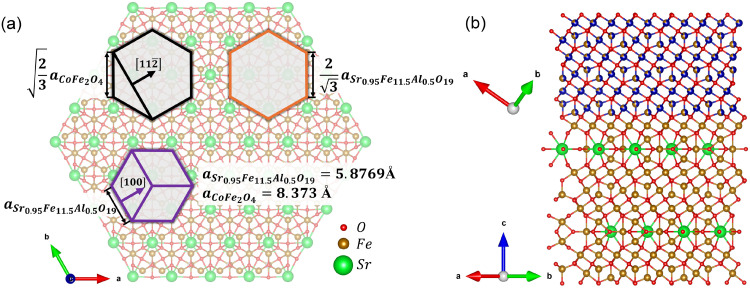
The proposed model of the interface between Co_1−*y*_Fe_2+*y*_O_4_ and Sr_0.95_Fe_11.5_Al_0.5_O_19_. Figure (a) depicts the atomic structure of the terminal layer of Sr_0.95_Fe_11.5_Al_0.5_O_19_, with the marked positions of the cobalt ferrite unit cell (black hexagon), hexaferrite unit cell (purple hexagon), and a unit of the coincide site lattice (orange hexagon). Lengths of hexagon sides are given in the lattice parameter units. Figure (b) depicts the cross-sectional view of the interface.^39^

Based on the results of XRD and TEM collected in this research, along with the knowledge acquired from our previous studies,^23,24,27^ we proposed a model for the epitaxial interface between Sr_0.95_Fe_11.5_Al_0.5_O_19_ and Co_1−*y*_Fe_2+*y*_O_4_ (Fig. 4) and calculated the lattice mismatch *ε* using the appropriate equation:
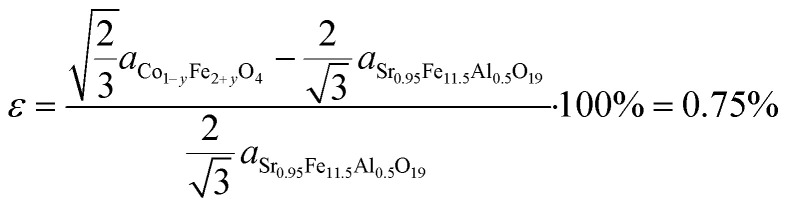


The algorithm proposed in work^41^ was employed to calculate the energy surface of the hexaferrite/cobalt ferrite interface and to determine its optimal configuration. Notably, the results of the *ε* calculation based on the proposed interface model align perfectly with the Rietveld refinement results of the XRD data. A positive *ε* value indicates that the CoFe_2_O_4_ nanolayer exerts tensile strain on the hexaferrite nanoplate. As the thickness of the CoFe_2_O_4_ layer increases, so does the strain, which is reflected in the increase of the hexaferrite *a* parameter. It is important to note that the parameter value used in modeling corresponds to that of bulk CoFe_2_O_4_ (*y* = 0). However, as discussed during the analysis of the EDX results, the spinel phase that has topotactically formed on SF nanoparticles contains an excessive amount of iron, suggesting that its bulk parameter should be greater than that of CoFe_2_O_4_ (because the ionic radius *r*^IV^(Fe^2+^) = 0.63 Å is larger than *r*^IV^(Co^2+^) = 0.58 Å). This implies an even greater compressive strain exerted on the spinel phase than indicated by the *ε* value.

**Fig. 5:**
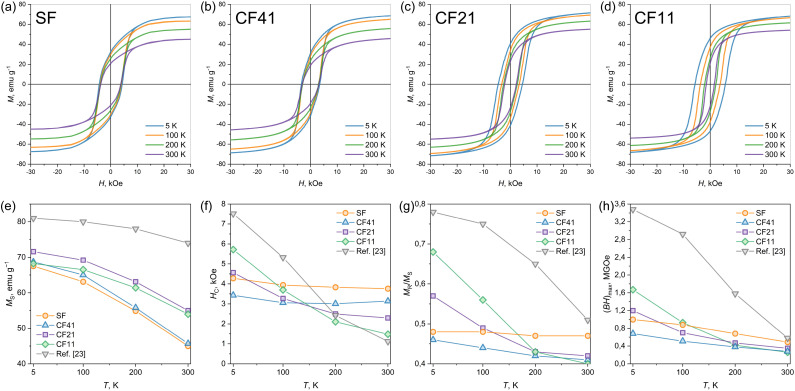
Hysteresis loops of initial Sr_0.95_Fe_11.5_Al_0.5_O_19_ nanoparticles SF (a), and hexaferrite/cobalt ferrite nanocomposites with 8 wt% (CF41), 48 wt% (CF21), and 55 wt% (CF11) of spinel phase acquired at different temperatures (b–d). Temperature dependencies of magnetic properties of the samples: saturation magnetization (e), coercivity (f), *M*_S_/*M*_R_ (g), maximum energy product (BH)_max_ (h). For comparison, data from work^23^ for a sample with a similar structure and a cobalt ferrite content of 85 wt% are also presented.

The results of magnetic measurements of bare Sr_0.95_Fe_11.5_Al_0.5_O_19_ nanoparticles and hexaferrite/cobalt ferrite nanocomposites are shown in Fig. 5 and summarized in Table 2. Bare Sr_0.95_Fe_11.5_Al_0.5_O_19_ nanoparticles demonstrate the hysteresis that is typical for the ensemble of unoriented particles with uniaxial magnetic anisotropy described by the Stoner–Wolfarth model: the *M*_R_/*M*_S_ is rather close to 0.5 and does not depend on temperature. The coercivity value at 300 K is 3760 Oe, and it increases slightly with the temperature decrease to 3830 Oe at 200 K, to 3940 Oe at 100 K, and finally to 4280 Oe at 5 K. It is known that hexaferrites, including those with a small substitution for aluminum, are characterized by a decrease in coercive force upon cooling.^33,42^ The fact that bulk strontium hexaferrite and its nanoparticles have the opposite trends of temperature dependency of coercivity is generally attributed to the contribution of the surface anisotropy, which rises due to the breaking of symmetry of the crystal field around iron ions that are located at the particle's surface.^43^

**Table 2:** Magnetic properties of the samples at different temperatures. *H*_C_ represents coercivity, *M*_S_—sample magnetization at 30 kOe, and *M*_R_—remanent magnetization

Sample	Temperature, K	300	200	100	5
SF	*H*_C_, Oe	3760	3830	3940	4280
	*M*_S_, emu g^−1^	45.0	54.9	63.1	67.5
	*M*_R_, emu g^−1^	21.1	25.8	30.2	32.3
	*M*_R_/*M*_S_	0.47	0.47	0.48	0.48
	(BH)_max_, MGOe	0.49	0.68	0.88	1.00
CF41	*H*_C_, Oe	3140	3010	3060	3430
	*M*_S_, emu g^−1^	45.7	55.8	65.0	68.7
	*M*_R_, emu g^−1^	18.8	23.6	28.4	31.3
	*M*_R_/*M*_S_	0.41	0.42	0.44	0.46
	(BH)_max_, MGOe	0.28	0.38	0.51	0.68
CF21	*H*_C_, Oe	2300	2500	3270	4560
	*M*_S_, emu g^−1^	55.0	63.1	69.2	71.6
	*M*_R_, emu g^−1^	23.1	27.2	33.8	40.9
	*M*_R_/*M*_S_	0.42	0.43	0.49	0.57
	(BH)_max_, MGOe	0.35	0.47	0.70	1.20
CF11	*H*_C_, Oe	1480	2110	3700	5720
	*M*_S_, emu g^−1^	54.0	61.4	66.5	68.2
	*M*_R_, emu g^−1^	21.6	26.6	37.5	46.5
	*M*_R_/*M*_S_	0.40	0.43	0.56	0.68
	(BH)_max_, MGOe	0.26	0.42	0.93	1.67

Hysteresis loops of hexaferrite/cobalt ferrite composites demonstrate the presence of a single bend, which means that the components are effectively exchange-coupled and behave as a single magnetic phase.^44,45^ This aligns with the results of estimations of spinel layer thickness: 0.2 nm in CF41, 2.8 nm in CF21, and 3.9 nm in CF11. As is known from the literature, the thickness of the domain wall in strontium hexaferrite is roughly 28 nm wide,^46^ and since the thickness of the spinel layer is significantly less, it should be fully involved in the exchange coupling. It also makes sense to compare the magnetic properties of the samples with sandwich composites possessing thicker outer layers of cobalt ferrite (85 wt% of CoFe_2_O_4_ and 15 nm layer thickness) obtained earlier by a similar method.^23^ The corresponding magnetic characteristics are also shown in Fig. 5e–h.

The saturation magnetization of the samples tends to increase with increasing proportion of cobalt ferrite. Deviation from this dependence when moving from sample CF21 to sample CF11 may be due to the presence of a small amount of unwashed non-magnetic contaminants (formed during the degradation of the organic environment) in the latter. At low temperatures, the magnetization of the samples naturally becomes higher, but with an increase in the proportion of cobalt ferrite, this enhancement becomes less pronounced. This is consistent with the fact that hexaferrite magnetization is more dependent on temperature.

At 300 K, the coercivity of the composite samples decreases with an increasing proportion of cobalt ferrite (Fig. 5f). Even a small addition of cobalt ferrite in the CF41 sample (approximately 7 wt%) causes a noticeable drop in coercive force from 3760 to 3140 Oe, and in samples with ferrite content of 50, 58, and 85 wt%, the room temperature coercivity reduces to 2300, 1480, and 1000 Oe, respectively. However, with decreasing temperature, the coercive force of the samples increases, and this effect is enhanced with increasing cobalt ferrite content. The coercivity of the sample with 50 wt% (CF21) exceeds the value for the initial hexaferrite particles below 50 K, with 58 wt% (CF11) – below 100 K, and with 85 wt%^23^ – below 150 K.

This temperature behavior is explained by the properties of the cobalt ferrite phase, in particular, its magnetic anisotropy. It is known that at room temperature, cobalt ferrite exhibits soft magnetic properties due to low magnetic anisotropy, and particles with a diameter of less than approximately 10 nm are completely superparamagnetic, that is, they have zero magnetic anisotropy.^14,47–50^ However, if the particle size does not exceed the single-domain limit (approximately 40 nm), upon cooling, the coercive force increases sharply, and at 5 K it reaches values of 16–20 kOe. A similar effect occurs in the studied composites: at room temperature, magnetic anisotropy is governed by hexaferrite cores, and soft magnetic cobalt ferrite reduces it, but upon cooling, the contribution of the cobalt ferrite layers becomes positive and determines the low-temperature magnetically hard properties. This is also confirmed by the observed effect of changing the *M*_R_/*M*_S_ ratio (Fig. 5g). At room temperature, thin layers of cobalt ferrite (samples CF41, CF21, and CF11), which do not have their own magnetocrystalline anisotropy (because for an autonomous phase particle of such size would have been superparamagnetic), introduce distortion into the uniaxial anisotropy of hexaferrite, lowering the ratio below 0.5. Thick layers (the sample from work^23^) already have their own cubic magnetocrystalline anisotropy (since they are larger than the superparamagnetic limit) and slightly increase the value of *M*_R_/*M*_S_ for the composite. As the temperature decreases, the contribution of cobalt ferrite to magnetic anisotropy becomes greater, so the ratio increases, since for a pure cobalt ferrite phase it should tend to approximately 0.8 (a typical squareness ratio for unoriented cubic crystals with three magnetic easy axes). The observed changes in coercivity, magnetization, and hysteresis shape led to regular changes in the magnetic energy product (BH)_max_ (Fig. 5h). The value of (BH)_max_ increases with increasing proportion of cobalt ferrite and decreasing temperature.

It is assumed that the exchange-coupled composite should synergistically combine the properties of the constituent phases. In metal–metal systems (rare-earth alloys^51^ or FePt-based materials^44^), the soft magnetic phase with higher magnetization increases the maximum energy product (BH)_max_. In ferrite–ferrite composites, both phases typically have close magnetizations, so other effects should be expected. As we have shown, in sandwiched Sr_0.95_Fe_11.5_Al_0.5_O_19_/Co_1−*y*_Fe_2+*y*_O_4_ composites, the properties change gradually from hexaferrite-like behavior to the characteristic behavior of cobalt ferrite with an increase in the proportion of the latter. The described synthetic approach allows modifying the properties of hexaferrite nanomagnets, and it can be extended to any spinel materials with suitable cell parameters, combining the properties of hexaferrite with various properties of the outer layers (magnetic, catalytic, optical, electrical, *etc.*). In the case presented here, the composite combines the high coercivity of cobalt ferrite at low temperatures with the hard magnetic behavior of hexaferrite near room temperature and its plate-like morphology.

## Conclusions

We have demonstrated that glass crystallized Al substituted hexaferrite nanoplates can be conformally coated on both basal faces with spinel cobalt ferrite layers by a single step thermolysis of metal acetylacetonates in hexadecane. Synthesis in a high-boiling solvent allows to reduce the aggregation of magnetic particles and to coat each particle individually, while maintaining the colloidal form of the resulting composite. We have shown that the phase ratio in the composites (and hence the thickness of the outer layers) can be tuned by changing the concentration of the starting solution. As a result, composites hexaferrite/cobalt ferrite with spinel phase content of 7, 50, and 58 wt% were obtained, which corresponds to average outer layer thicknesses of 0.2, 2.8, and 3.9 nm. The outer layers in the resulting sandwich-like nanoparticles grow epitaxially, continuing the crystalline structure of the hexaferrite nanoplatelets, so the composites demonstrate effective exchange-coupling between the constituent phases. With an increase in the proportion of the spinel phase, the characteristics of the composites change gradually, moving from hexaferrite-like behavior to properties typical of cobalt ferrite nanoparticles.

Our single-step, gram-scale thermolysis route yields tunable epitaxial spinel layers on strontium hexaferrite nanoplates and, for the first time, maps magnetic performance as a function of phase content. The proposed approach is universal, allowing for the most efficient integration of hexaferrite nanoparticles with other phases that have matching lattice parameters. These outer layers can provide additional capabilities due to their magnetic, electronic, optical, catalytic, and other properties. At the same time, hexaferrite cores will give the composite particles the properties of nanomagnets, whose position, orientation, as well as rotational and translational motion, can be controlled by external magnetic fields. Thus, this research is yet another step towards the development of composite colloidal nanomagnets that combine the properties of different magnetic and non-magnetic phases.

## Author contributions

Conceptualization, E. O. A., E. A. G. and L. A. T.; formal analysis, E. O. A., E. A. G. and L. A. T.; investigation, R. N., A. N. V., J. C., E. O. A., E. S. K., M. S. K. and S. V. T.; methodology, E. O. A., E. A. G. and L. A. T.; project administration, L. A. T.; resources, E. S. K., M. S. K. and S. V. T.; supervision, L. A. T.; visualization, R. N., E. O. A., M. S. K. and S. V. T.; writing – original draft, R. N., A. N. V., E. O. A., E. A. G. and L. A. T; writing – review & editing, E. A. G. and L. A. T. All authors have read and approved the decisive version of the manuscript.

## Conflicts of interest

There are no conflicts to declare.

## Data Availability

Data for this article, including XRD, TEM statistics, and VSM are available on osf.io (OSF) *via* DOI https://doi.org/10.17605/OSF.IO/7YFKZ.
